# Reliability of residents’ assessments of their postgraduate medical education learning environment: an observational study

**DOI:** 10.1186/s12909-019-1874-6

**Published:** 2019-12-03

**Authors:** Paul L. P. Brand, H. Jeroen Rosingh, Maarten A. C. Meijssen, Ingrid M. Nijholt, Saskia Dünnwald, Jelle Prins, Johanna Schönrock-Adema

**Affiliations:** 1 Isala Academy, Department of Medical Education and Faculty Development, Isala Hospital, Zwolle, the Netherlands; 2 Center for Education Development and Research in Health Professions, University of Groningen and University Medical Centre, Groningen, the Netherlands; 3 Department of Ear, Nose and Throat Surgery, Isala Hospital, Zwolle, the Netherlands; 4 Department of Gastroenterology, Isala Hospital, Zwolle, the Netherlands; 5 MCL Academy, Medical Center Leeuwarden, Leeuwarden, the Netherlands

**Keywords:** Learning environment, SPEED, Postgraduate medical education, Quality cycle

## Abstract

**Background:**

Even in anonymous evaluations of a postgraduate medical education (PGME) program, residents may be reluctant to provide an honest evaluation of their PGME program, because they fear embarrassment or repercussions from their supervisors if their anonymity as a respondent is endangered. This study was set up to test the hypothesis that current residents in a PGME program provide more positive evaluations of their PGME program than residents having completed it. We therefore compared PGME learning environment evaluations of current residents in the program to leaving residents having completed it.

**Methods:**

This observational study used data gathered routinely in the quality cycle of PGME programs at two Dutch teaching hospitals to test our hypothesis. At both hospitals, all current PGME residents are requested to complete the Scan of Postgraduate Education Environment Domains (SPEED) annually. Residents leaving the hospital after completion of the PGME program are also asked to complete the SPEED after an exit interview with the hospital’s independent residency coordinator. All SPEED evaluations are collected and analysed anonymously. We compared the residents’ grades (on a continuous scale ranging from 0 (poor) to 10 (excellent)) on the three SPEED domains (content, atmosphere, and organization of the program) and their mean (overall department grade) between current and leaving residents.

**Results:**

Mean (SD) overall SPEED department grades were 8.00 (0.52) for 287 current residents in 39 PGME programs and 8.07 (0.48) for 170 leaving residents in 39 programs. Neither the overall SPEED department grades (t test, *p* = 0.53, 95% CI for difference − 0.16 to 0.31) nor the department SPEED domain grades (MANOVA, F(3, 62) = 0.79, *p* = 0.51) were significantly different between current and leaving residents.

**Conclusions:**

Residents leaving the program did not provide more critical evaluations of their PGME learning environment than current residents in the program. This suggests that current residents’ evaluations of their postgraduate learning environment were not affected by social desirability bias or fear of repercussions from faculty.

## Background

In postgraduate medical education (PGME), a department’s learning environment is considered to be vital for high-quality postgraduate medical education [1, 2]. A healthy learning environment is associated with improved resident well-being [3, 4], a reduced risk of resident burnout [5, 6], and better preparedness for practice after completing residency [7]. As a result, a healthy learning environment may not only support the professional development of residents but also improve the quality of the patient care they provide [8].

The learning environment has been described as the formal and informal context in which learning takes place [8], comprising the content, atmosphere and organization of the education program [2, 9]. The Scan of Postgraduate Education Environment Domains (SPEED) was developed and validated as a concise instrument, based on a solid theoretical framework [2], to capture residents’ perceptions of these three domains of PGME programs [9]. In the absence of a gold reference standard for the quality of the learning environment, the residents’ assessment of the learning environment is generally accepted as the most important tool in the quality cycle of PGME programs [10–12].

Residents generally feel capable of assessing the quality of their PGME program and providing feedback on it to their supervisors [13]. Anonymizing residents’ evaluations of the PGME learning environment is considered desirable by both residents and instrument developers to increase the likelihood of obtaining an accurate and honest assessment of the learning environment [14–18]. Even if a PGME program evaluation instrument is applied as a web-based survey without disclosing respondent identity, residents remain concerned about their anonymity [16, 17, 19]. They express reluctance to reveal their honest opinions regarding their PGME program when they are dependent on their supervisor for summative assessments or will be involved in future interactions with the supervisors they have to evaluate [16, 20], particularly if they think their responses can be traced back to them personally. The perceived risk of such identity disclosure is likely to be larger in smaller departments with fewer residents [19]. Therefore, we hypothesised that the dependency issue with the associated fear of repercussions applies more to current residents in the PGME program than to residents who leave the PGME program after having completed it. The aim of this study was to assess whether leaving residents report lower learning environment scores than current PGME residents, and whether this was more likely to occur at departments with fewer residents, where perceived respondent anonymity may be at risk.

## Methods

### Setting

In the Netherlands, PGME programs consist of 4–6 years of workplace learning in teaching hospitals, partly in a general teaching hospital and partly in a university hospital. Competition for enrolment in nationally recognized PGME programs is fierce, which is why the majority of freshly graduated doctors choose to obtain clinical experience for a few years as a junior doctor before applying for a residency position. As a result, almost all PGME departments in Dutch teaching hospitals employ both junior doctors not enrolled in formal PGME training, and residents in the nationally recognized PGME program of that discipline. Both junior doctors and residents are licensed physicians and are involved in patient care, with residents acting increasingly independently with increasing experience and competence throughout the PGME program. Although junior doctors are not formally enrolled in PGME programs, they participate in the department’s educational activities for residents and share clinical duties and on-call shifts with residents.

We conducted a cross-sectional observational study of residents and junior doctors in two hospitals in the Netherlands: Isala Hospital in Zwolle (1100 beds) and the Medical Center in Leeuwarden (MCL, 618 beds). Isala and MCL employ approximately 120 and 95 residents in formal PGME programs and 100 and 65 junior doctors, respectively. Both hospitals are certified by the Royal Dutch Medical Association as licensed general teaching hospitals in 28 and 23 PGME programs, respectively. Because each PGME program has its own design and timetable, the population of residents in Isala and MCL changes almost every month, with residents moving in and out of PGME programs. Residents spend between 6 and 48 months of their PGME training at the hospital, depending on the program they are enrolled in.

### Quality cycle of PGME programs

As prescribed by the Royal Dutch College of Medicine [21], both Isala and MCL hospitals carry out an quality cycle, aimed at continuously monitoring and improving the quality of each PGME program. As part of this quality cycle, all current residents and junior doctors are asked to complete the SPEED questionnaire annually by web-based survey, the results of which are analysed and fed back to faculty anonymously (i.e, without disclosing individual respondents’ responses or characteristics).

Each resident or junior doctor leaving the hospital after completion of the PGME program (resident) or expiration of their contract (junior doctor) is invited for an exit interview, collecting data on the resident’s or junior doctor’s experience in working at the hospital in a semi-structured fashion. The aggregated results of these exit interviews are fed back to faculty, again without disclosing individual respondents’ responses or characteristics. These exit interviews are being conducted by the hospital’s junior staff coordinators, who are the primary contact persons for residents and junior doctors throughout their career at the hospital, and who are independent from the hospital’s faculty providing the PGME programs. These junior staff coordinators are highly valued by residents and junior doctors as their advocates and confidants, and serve the recommended role as an independent “honest broker” to collect anonymous data on PGME program quality [16]. As part of the exit interview, residents and junior doctors are asked to complete the SPEED by web-based survey.

### Study population and outcome measures

We used the SPEED results that were collected routinely as part of the PGME quality cycle in the two hospitals, in two groups of residents and junior doctors:

- Residents and junior doctors currently working at the hospital (called “current residents” in the remainder of this article)
- Residents and junior doctors participating in an exit interview as outlined above (called “leaving residents” in the text below)

Between January and December 2017, all 220 current residents at Isala were invited to complete the web-based SPEED survey. At the MCL, all 160 current residents were asked to complete the web-based SPEED survey between October 2017 and October 2018.

Throughout 2017, exit interviews were conducted with all 95 leaving residents at Isala and with all 75 residents leaving MCL in 2018.

### Speed

The SPEED comprises 15 items in three domains (content, atmosphere, and organisation of the PGME program), scored on a 5-point Likert scale ranging from one (strongly disagree) to five (strongly agree), and a *general domain grade* for each domain on a scale from 1 (poor) to 10 (excellent) [9]. This way of general grading is used throughout secondary and university education in the Netherlands and is therefore familiar to residents. The SPEED is completed in a web-based survey. Responses are collected anonymously; no data are recorded on age, gender or other personal characteristics, apart from the department at which the respondents are working.

The full version of the SPEED is available in its original open access publication [9].

### Statistical analysis

Because of the anonymity of the data, we were not able to link individual scores of current residents to those of leaving residents. Our analyses were based on the following variables for each department:

- **department SPEED domain grades**: we calculated three *department SPEED domain grades* by averaging per department the *general domain grades* given by respondents for content, atmosphere, and organization of the program;
- **overall department SPEED grade**: for each respondent, we calculated the *mean SPEED grade* by averaging the three *general domain grades*; subsequently, we calculated the *overall department SPEED grade* by averaging the *mean SPEED* grades per department.

Because the distributions of the department SPEED domain grades and the overall department SPEED grades were not significantly different from normal distributions (Kolmogorov-Smirnov tests, *p* > 0.1) we used parametric tests (MANOVA and Student’s t tests) to analyse the data.

We used multivariate analysis of variance (MANOVA) to assess differences in our primary outcome parameter, i.e. the department SPEED domain grades, between current and leaving residents. We also examined differences in the primary outcome parameters between the two study sites, to explore potential systematic differences in perceived learning environment quality between hospitals. We used Student’s *t* test to analyse the difference in overall department SPEED grades between current and leaving residents and between hospitals.

As secondary outcome parameters, we analysed whether the differences in SPEED domain grades and overall department SPEED grades between current and leaving residents were related to the number of residents in a department, by comparing it between large departments (> 5 residents) and small departments (< 5 residents), and by calculating the correlation coefficient between the number of residents in a department and the difference in department SPEED grades between current and leaving residents.

Before the study, we considered that a 1 point difference between department SPEED grades of current and leaving residents represented a relevant difference in the residents’ assessment of their learning environment. To be able to detect such a difference with 90% power, assuming a SPEED grade standard deviation of 0.5 [9], we needed to compare SPEED scores between current and leaving residents of at least 12 departments.

All analyses were carried out using IBM SPSS statistics.

### Ethical considerations

This study was approved by the Netherlands Association for Medical Education Ethical Review Board (file number 1063).

## Results

### Response rate

Completed SPEED questionnaires were obtained from 193 current residents in the program at 21 departments at Isala (response rate 88%) and from 96 current residents in 18 programs at MCL (response rate 60%). Exit interviews including SPEED domain grades were completed by 95 leaving residents from 21 departments at Isala and by 75 leaving residents from 18 departments at MCL (response rate at both hospitals 100%). There were no significant differences between hospitals in the department SPEED domain grades (MANOVA, F(3,74) = 1.25, *p* = 0.30) or the overall department SPEED grades (*t* test, *p* = 0.29).

### Primary outcome parameter

**Fig. 1:**
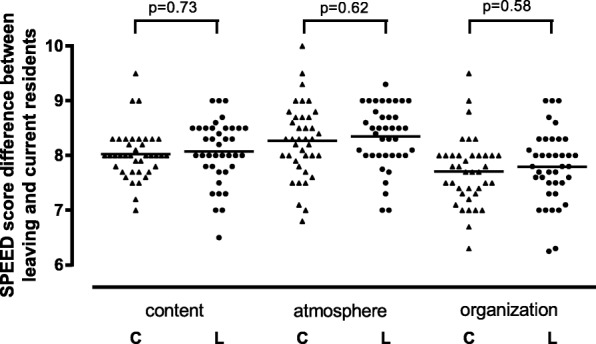
Department SPEED domain grades for the content, atmosphere and organization of the PGME program as provided by current residents (C, triangles) and leaving residents (L, circles). Bars represent means. MANOVA showed no significant differences in department SPEED domain grades between current and leaving residents (see text)

Department SPEED domain grades for the content, atmosphere and organization of the PGME program were comparable between current and leaving residents (Fig. 1). There were no significant differences in these SPEED domain grades (MANOVA, F(3, 62) = 0.79, *p* = 0.51) or the overall department SPEED grades between current (mean 8.00, SD 0.52) and leaving residents (mean 8.07, SD 0.48, 95% CI for difference − 0.16 to 0.31, *p* = 0.53).

### Secondary analyses

There was a trend towards higher department SPEED domain grades in residents from smaller teaching departments than in those from larger teaching departments, but these differences only reached marginally statistical significance for the organization domain scores of current residents (Table 1). There was a significant, positive correlation between the number of residents in a department and the difference in overall department SPEED grades between current and leaving residents (r = 0.361, *p* = 0.026), with leaving residents in larger departments providing lower grades than current residents. In large departments, the difference in overall SPEED department grades between current and leaving residents was slightly larger than in small departments (95% CI for difference 0.03–0.63, *p* = 0.03, see Table 2). This difference was completely explained by the organization domain (Table 1).

**Table 1:** Comparison of department SPEED domain grades and overall department SPEED grades between residents from departments with < 5 or > 5 residents

SPEED domain	Residents from the 23 small departments with < 5 residents		Residents from the 16 large departments with > 5 residents		P*	95% CI for difference
	Mean	SD	Mean	SD		
Current residents (*n* = 289)						
Content	8.13	0.51	7.88	0.34	0.101	−0.54 to 0.05
Atmosphere	8.39	0.77	8.09	0.46	0.166	−0.74 to 0.13
Organization	7.88	0.68	7.47	0.43	0.039	−0.80 to − 0.02
Overall score	8.13	0.58	7.81	0.34	0.057	−0.65 to 0.01
Leaving residents (*n* = 170)						
Content	8.11	0.59	8.01	0.56	0.597	−0.48 to 0.28
Atmosphere	8.42	0.53	8.24	0.63	0.349	−0.55 to 0.20
Organization	7.67	0.65	7.98	0.62	0.148	−0.11 to 0.73
Overall score	8.07	0.51	8.08	0.45	0.948	−0.31 to 0.33

* independent samples *t* test

**Table 2:** difference in mean SPEED domain grades between leaving and current residents, compared between small (< 5) and large departments (> 5 residents)

SPEED domain	Departments with < 5 residents (*n* = 23)		Department with > 5 residents (*n* = 16)		P*	95% CI for difference
	Mean difference	SD	Mean difference	SD		
Content	−0.02	0.52	0.13	0.45	0.366	−0.18 to 0.46
Atmosphere	0.03	0.71	0.15	0.48	0.532	−0.28 to 0.54
Organization	−0.21	0.68	0.51	0.64	0.002	0.28 to 1.16
Overall score	−0.06	0.51	0.27	0.35	0.033	0.03 to 0.63

* independent samples *t*-test

## Discussion

In this study of residents from two general teaching hospitals in the Netherlands, we found no statistically significant differences in overall department SPEEED grades or department SPEED domain grades between residents leaving the PGME program and residents currently enrolled in the program (Fig. 1). This study thus showed that residents leaving the program did not provide more critical evaluations of their PGME learning environment than current residents in the program.

We considered several potential explanations for this finding. First, and most likely, the current residents in both teaching hospitals may have felt safe enough to provide the organization with honest feedback on their PGME learning environment. Second, differences between the cohorts of current and leaving residents may have distorted the outcomes. However, although individual experiences of a PGME program likely differ between residents, there are no clear reasons to expect systematic differences in experiences of the PGME program between current and leaving residents. They followed the same program, with the same supervisors, performed the same clinical work and followed the same formal education sessions outside the clinical workplace. In addition, the cohorts of current and leaving residents overlapped in part. During the study, there were no interventions targeted at improving or changing the learning environment in the two hospitals that may have affected our findings. Moreover, research on data of 7 cohorts of medical students showed that differences between cohorts explained only 0.01% of the variance in multiple choice examination results, compared to 83% for the differences between subjects within cohorts, and 12% for random error [22]. Similarly, research among residents showed that repeated learning environment assessments by different groups of residents for quality assurance and improvement purposes did not show any meaningful changes in overall scores over time [8].

Third, leaving residents’ SPEED scores could have been affected by social desirability bias, if these residents desired to stay at or return to the same department later in their career. However, it is unlikely that this would apply to all leaving residents. In addition, even if residents who wish to stay provided higher SPEED scores, it is unknown whether this reflects social desirability bias or true satisfaction with the program. Fourth, considering that leaving residents would benefit less from any improvements to the PGME program based on their critical feedback, leaving residents may be subject to the so-called “peak end” effect, i.e. the tendency of people to evaluate experiences based on the best or worst components at the end of the experience rather than comprehensively [23]. We had no reason to believe that leaving residents refrained from providing open and honest feedback, however, as the exit interviews were collected by independent “honest brokers” [16].

The study was sufficiently powered to detect a relevant difference in learning environment scores between current and leaving residents, making it unlikely that a larger study would have shown significant differences in SPEED grades between these two groups of residents.

To our knowledge, our study is the first to compare evaluations of PGME programs between current and leaving residents. Our findings argue against bias in current residents’ evaluation of the quality of their PGME learning environment. The trend towards higher SPEED grades in small departments, and towards larger differences in SPEED domain grades between current and leaving residents in larger departments was completely explained by the organisation domain, and was in the opposite direction than expected if current residents were concerned of identity disclosure with the associated fear of embarrassment or repercussions from their supervisors [16, 20]. This is reassuring given the importance of these assessments in the quality control and management of PGME programs. It has been suggested that residents’ evaluations of the learning environment are less susceptible to social desirability bias than residents’ evaluations of individual supervisors [16], suggesting the need for further studies to compare individual supervisors’ evaluations between current and leaving residents.

The strengths of this study include the use of a validated concise tool with a sound theoretical basis to assess the learning environment [2, 9], the setting of two large general teaching hospitals with a wide range of PGME programs and resident numbers, comprising medical, surgical and supportive disciplines, and the high response rate. The main limitation of our study is that the requirement of respondent anonymity made it impossible to analyse differences between junior doctors and residents enrolled in PGME programs, or between residents with different years of completed PGME training. It also precluded the ability to directly compare individual residents’ evaluations of their PGME program as current and as leaving residents. The ideal study design to address our research question would be a longitudinal cohort study of residents followed up throughout residency and after completing it. However, the long-term nature of such a study could increase the participants’ (perceived) risk of identity disclosure which would undermine its advantages, either by reducing the residents’ willingness to participate in the study or by introducing social desirability bias. The Netherlands has a unique system of hospital-wide education committees supervising the quality of residency training [24], which may have contributed to the residents in this study feeling free to provide an unbiased assessment of their department’s learning environment. Further studies are needed to examine the impact of social desirability and potential other biases on resident’s assessment of the learning environment in other settings and countries. Qualitative studies might offer alternative opportunities to find out whether residents feel free to evaluate their PGME programs honestly and which barriers they perceive to do this.

## Conclusion

We found comparable evaluations of the PGME learning environment between residents having completed the program and residents in the program. We argued that there was no effect of social desirability bias on these evaluations, and that the outcomes of these evaluations by residents currently enrolled in the program seem trustworthy.

## Data Availability

The SPEED items are available through its open access original publication [9]. The datasets used and/or analysed during the current study available from the corresponding author on reasonable request.

## Abbreviations

MANOVA: multivariate analysis of variance
MCL: Medical Center Leeuwarden
PGME: postgraduate medical education
SD: standard deviation
SPEED: Scan of Postgraduate Educational Environment Domains
